# The Economic Costs of Informal Care: Estimates from a National Cross-Sectional Survey in The Netherlands

**DOI:** 10.1007/s10198-023-01666-8

**Published:** 2024-01-31

**Authors:** Saif Elayan, Viola Angelini, Erik Buskens, Alice de Boer

**Affiliations:** 1 Department of Economics, Econometrics and Finance, Faculty of Economics and Business, University of Groningen, Nettelbosje 2, 9747 AE Groningen, The Netherlands; 2 Department of Epidemiology, University Medical Centre Groningen, University of Groningen, Hanzeplein 1, 9700 RB Groningen, The Netherlands; 3 Faculty of Social Sciences, Vrije Universiteit Amsterdam, 1081 HV Amsterdam, The Netherlands; 4 The Netherlands Institute for Social Research (SCP), 2500 BD The Hague, The Netherlands

**Keywords:** Informal care, Economic costs of care, Cost analysis, Indirect costs, Opportunity cost, Out-of-pocket expenses, I11, I15, I18, J14, J22

## Abstract

Faced with an unprecedented demand for long-term care, European health care systems are moving towards mixed care models, where the welfare state and informal caregivers share care responsibilities. While informal care is often viewed as a means of alleviating pressure on public care, it comes with significant economic costs for caregivers, their employers, and society at large. This study uses nationally representative data to estimate the total direct (informal care time and out-of-pocket costs) and indirect (productivity) economic costs of informal care in the Netherlands in 2019. Informal care time costs are estimated using the opportunity cost and the proxy good methods. Indirect costs are estimated using the human capital and friction cost approaches. Our results reveal the considerable annual societal cost of informal care in the Netherlands, ranging between €17.5 billion and €30.1 billion, depending on the valuation approach. These costs are equivalent to 2.15% and 3.71% of Dutch GDP in 2019, comparable to the public expenditure on long-term care in that year. Female caregivers account for slightly more than half (53%–57%) of the total costs. Around 57%–88% of these costs are in the form of informal care time. The main driver of indirect costs is the temporary cessation of work, which comprises 12%–17% of the total costs. Findings corroborate that substantial resources, yet thus far largely disregarded, are spent on informal care even in a country with a relatively generous public long-term care system.

## Introduction

Since the 1960s, the demographic landscape of Europe has undergone significant changes. The most notable transformation has perhaps been the ageing of the population, driven by the combined effects of declining fertility rates and increasing life expectancy [1, 2]. This trend is reflected in the old-age dependency ratio, which has more than doubled from 16.1% in 1960 to 33.5% in 2020 [3]. Currently, nearly one-fifth of the European Union population is 65 or older, a proportion projected to rise to 30.9% by 2050, with the fastest growth anticipated in the 80 + age category [4]. While population ageing is in many ways a success story, it poses significant challenges to the sustainability of public services such as pensions, health care, and long-term care (LTC).

In response to the escalating demand for LTC, European countries have enacted diverse policy reforms. Despite this diversity, a prevalent trend is discernible: a contraction in eligibility for and the scale of institutional care, accompanied by a growing reliance on informal care [5]. This policy shift is primarily predicated on the premise that informal care can serve as a cost-saving strategy, efficiently addressing the growing demand for LTC while simultaneously curbing the associated costs [6]. One critique of this cost-saving notion is that it overlooks the already substantial amount of informal care being provided, thus overestimating the potential for “untapped” informal care [7]. This critique finds empirical corroboration from the 2016 European data, which indicates that nearly 13% of the population, over 76 million people, actively engaged in informal caregiving, collectively amassing an astounding 72 billion hours of care [8]. Concerns also persist regarding the future availability of informal care. Ribeiro et al. project that the caregiver support ratio, defined as the number of individuals aged 45–64 available to provide care for each older adult aged 80 + , will steadily decrease throughout Europe in the coming decades. Specifically, by 2050, this ratio is estimated to drop to just two potential informal caregivers for each older adult, significantly down from the approximately five-to-one ratio observed in 2020 [9].

This notion of cost savings is also criticised for disregarding the negative externalities of informal care, including the expenses incurred by informal caregivers, their employers, and society as a whole [7]. Fast et al. denote these costs as the “hidden costs” of informal care and formulate a taxonomy that divides them into two primary categories: economic and non-economic costs [10]. The latter, termed intangible costs, arise from potential declines in the physical, social, and emotional well-being of informal caregivers. Furthermore, the taxonomy identifies three main sources of economic costs associated with informal care, including employment-related costs, such as foregone employment opportunities or diminished productivity; out-of-pocket expenses that caregivers would not incur without providing care; and caregiving labour, which encompasses the time devoted by informal caregivers to providing care. In the field of health economics, employment-related costs are conventionally classified as indirect (productivity) costs, while caregiving labour and out-of-pocket costs are commonly classified as direct costs [11].

The inclusion of informal care costs in health economic evaluations and cost-of-illness studies has recently gained traction, but it remains an uncommon practice [12]. Several scholars caution against the negative consequences of omitting these costs when evaluating policies and making resource allocation decisions. For instance, a systematic review of economic evaluations in areas where informal care is significant, conducted by Krol et al., demonstrates the significant impact that including informal care costs can have on the outcomes of these evaluations [13]. This impact is likely to be even more pronounced in economic evaluations of support services and technologies for caregivers, such as training programmes and caregiver robots [14]. These concerns are echoed by van den Berg et al., who argue that ignoring costs related to informal care in policy- and decision-making can result in an “invisible” shift of costs from the LTC sector to the informal economy without ever realising real cost savings for society [15].

This paper contends that the assertion of cost containment or savings concerning the ongoing policy shift towards informal care cannot be substantiated without carefully considering the costs associated with informal care. We examine the Netherlands, a country transitioning from an LTC system with a traditionally high residential care rate to an ageing-in-place model that increasingly depends on informal caregivers. This study aims to estimate the total societal economic costs of informal care in the Netherlands for 2019. These estimates will unveil the societal value of the significant yet often overlooked informal care resource—a value consistently omitted from national income accounts and mainstream economic discourse. This information can provide policymakers with a nuanced understanding of the multifaceted impacts of LTC policies across the entire care sector. Furthermore, this study offers essential insights into the dynamics between informal care and the labour market. These insights can inform the development of mechanisms that help caregivers balance paid employment with caregiving duties. Lastly, as global reliance on informal care grows, our findings promise to contribute to discussions in other countries grappling with the challenge of meeting the burgeoning demand for LTC.

## Review of the current evidence on the costs of informal care

Our literature review identified several studies estimating the national cost of informal care. In the United States, Chari et al. estimated the cost of informal care for older adults in 2012 to be in the range of US$221 billion to US$642 billion, corresponding to 1.36% to 3.95% of US GDP [16, 17]. The economic value of informal care in Australia in 2020 was estimated by Deloitte Access Economics to be between A$15.2 billion, using the opportunity cost method, and A$77.9 billion, using the proxy good method, equating to 0.80% to 4.00% of Australia’s GDP [18].

In Spain, Oliva-Moreno et al. determined that in 2013, adult informal caregivers provided an average of 96.6 h per week, with the annual cost per caregiver ranging from €27 140, using the opportunity cost method, to €83 056, using the proxy good method. However, the study’s reliance on a small sample from two Spanish regions could limit its national representativeness [19]. In Sweden, the cost of informal care in 2019 was estimated by Ekman et al. at 3.15%–4.01% of the country’s GDP, notably including presenteeism and lost sleep due to informal care as cost components [20].

Ecorys Netherlands estimated the 2019 cost of informal care in the Netherlands to be between €22 billion, using the opportunity cost method, and €32 billion, using the proxy good method. The report, however, relied exclusively on data from published reports and articles, which lacked sufficient detail to capture the full scope of indirect (productivity) costs [21]. In Ireland, Hanly and Sheerin valued the informal care provided in 2011 at €5.3 billion (opportunity cost method), equivalent to 3.30% of the country’s GDP, with female caregivers accounting for almost two-thirds of the total cost [22, 23]. In a broader European context, Peña-Longobardo and Oliva-Moreno valued informal care in Europe in 2016 using data from the European Quality of Life Survey at approximately €576 billion, representing about 3.63% of Europe’s GDP [8].

These findings underscore the significant economic impact of informal caregiving, an apparent trend across countries with diverse LTC systems. Moreover, we observe considerable methodological variations, resulting in conspicuous discrepancies in the outcomes. These disparities underscore the non-neutral role of the chosen valuation methods and the specific cost components considered. The present study enhances existing literature by leveraging nationally representative individual-level data to precisely and comprehensively quantify the direct and indirect (productivity) costs of informal care. Acknowledging the impact of methodological choices, we employ the most commonly used methods for valuing informal care time and indirect (productivity) costs—namely, opportunity cost, proxy good, and contingent valuation methods for the former, and human capital and friction cost approaches for the latter. This strategy aligns our study with the predominant methodologies in caregiving research and significantly contributes to the field by providing relevant and comparable findings.

## Methods

### Data and variables

The primary data source used in this study comes from the third wave of the Informal Care (IZG) Study, a nationally representative survey examining the provision of informal care among the noninstitutionalised population aged 16 years or older residing in the Netherlands. The study was conducted between September 10, 2019, and January 10, 2020, by Statistics Netherlands on behalf of the Netherlands Institute for Social Research. The survey was carried out among a random sample of 38 923 subjects drawn from the municipal personal records database that includes all residents of the Netherlands. A total of 12 348 participants completed the survey, with 71% of the responses collected through a web-based survey and the remaining 29% obtained through telephone interviews. Survey weights were developed by Statistics Netherlands to account for unequal probability of selection and non-response, thus allowing for estimates representative of the underlying target population. More details on the sampling methodology are available elsewhere [24].

The survey commenced with demographic and household composition questions. Next, participants were presented with a definition of informal care (without specifying the term) and were then asked if they had provided this type of support in the previous 12 months. The definition was: “Providing help to friends or family with health problems. These can, for example, include your partner, family members, friends or neighbours who need help due to physical, psychological or mental limitations, or old age. Examples of such help include household activities, washing and dressing, companionship, transport and odd jobs. Help provided within the framework of your profession or volunteer work does not count in this regard” ([25], p. 1782). This definition has three advantages. First, it is broad enough to encompass the full range of informal caregivers, including those who do not recognise themselves as such, without imposing minimum thresholds on the amount or duration of care provided. Second, it clearly distinguishes between informal care on one side and volunteer and professional care on the other. Third, it excludes care provided to healthy individuals (e.g., child care). Participants identified as informal caregivers were then directed to a series of survey sections, each addressing a distinct aspect of informal care. The survey questions used in the costing analysis are presented in Table 1.

**Table 1:** List of survey questions used to estimate informal care costs

Cost component assessed	Survey question	Answer choices
A. Direct costs		
A.1 Informal care time	On average, how many hours per week do you spend assisting the person you provide help to? Please exclude any time spent on normal housework or the usual support that family/household members provide to each other	Number in the range 1–168
A.2 Out-of-pocket costs	Did you incur any additional costs associated with providing assistance? For example, travel or telephone costs	Yes/No
	Adding these costs together, how much did you spend in euros in the last month?	• Less than €50
		• Between €50 and €100
		• Between €100 and €200
		• Between €200 and €300
		• Greater than €300
B. Indirect costs (productivity loss)		
B.1 Cessation of work	Did you stop working to provide help?	Yes/No
	Have you stopped working in the past 12 months to provide help?	Yes/No
	Have you stopped working temporarily in the past 12 months to provide help?	Yes/No
B2. Reduction of work hours	Have you reduced your working hours in the past 12 months to provide help?	Yes/No
B3. Presenteeism (lost productivity while at work)	Did you work with less concentration due to the help you provided?	• No, not at all
		• Yes, a bit
		• Yes, very much
	How often were you distracted at work by the person you provided help to or by things you had to arrange for them?	• Daily
		• Weekly
		• Monthly
		• Rarely or never

### General approach

The present study estimated the economic cost of informal care from a societal perspective, where all relevant costs were considered, irrespective of whom they fall upon. We identified two broad domains of societal economic costs associated with informal care: direct and indirect (productivity) costs [10, 26]. Direct costs included the cost of time devoted to informal care and the out-of-pocket expenses incurred by caregivers [11]. Indirect (productivity) costs encompassed losses in work productivity due to care-related absenteeism and presenteeism [27].

Economic costs of informal care were estimated using a bottom-up and prevalence approach. The estimation proceeded in two steps. The first step involved measuring the quantities of resources used (i.e., informal care time and out-of-pocket expenses) and lost productivity (i.e., lost paid-work time). Data on resource use and lost productivity were obtained from the Informal Care Study (see Table 1). The second step involved valuing these resources and productivity losses by assigning unit costs. Costs were then estimated by multiplying the quantities of these resources and losses by their respective unit costs. Unit costs were derived or estimated from official sources, published literature, or the Dutch Manual for Costing Studies in Health Care [28]. A full list of unit costs and their sources is provided in Table 6 in Appendix 1. All prices and unit costs were adjusted to 2019 prices using the Consumer Price Index (CPI) [29].

Finally, a base-case analysis was developed using the best-available information and by applying the most conservative assumptions when data were lacking. Discounting of costs was not required since the time horizon was limited to one year. Weighted data were used in all analyses to obtain population estimates. All analyses were performed using the R software version 4.2.2 [30].

### Cost calculations

#### Direct costs

The weekly cost of informal care time was calculated for each caregiver as the product of the reported weekly hours of informal care and its unit cost. This estimate was then multiplied by the reported number of weeks of care provided in the past year to obtain the annual cost.

The unit cost of informal care time was determined by assigning a shadow price based on the opportunity cost and proxy good methods. In the opportunity cost method, informal care time was valued in terms of leisure time forgone. Thus, the unit cost was set equal to the value of one hour of leisure time. The value of leisure time can be determined as a proportion of the national average gross wage or via methods such as conjoint analysis, contingent valuation, and studies on the value of travel time savings. Given the lack of agreement on a standardised approach for valuing leisure time, and in line with the conservative approach applied in our base-case analysis, we selected the most conservative estimate available for the Netherlands. Consequently, we set the value of one leisure hour at the monetary equivalent of one hour of non-business travel time saving, as derived from the official monetary values of time published by the Dutch Ministry of Infrastructure and the Environment [31]. By contrast, in the proxy good method, informal care time was valued at the labour market price of a close substitute [32]. Following recommendations from the Dutch Costing Manual, the unit cost of informal care time was set equal to the standard hourly rate of household care (“huishoudelijke zorg” in Dutch) [28].

Previous research has shown that caregivers may report implausible hours of caregiving. Specifically, caregivers of severely dependent individuals may indicate that they provide continuous care, 24 h per day and seven days per week, due to the need to be constantly available for their care recipients [33]. However, this is obviously implausible, as caregivers must also allocate time to fulfil their basic needs, such as personal care and sleep [34]. Therefore, we imposed a minimum of 6 h per day for leisure, resulting in a maximum of 126 h per week available for work and informal care, calculated as (24 h—6 h) × 7 days. In cases where the combined time spent on work and care exceeded 126 h per week, the surplus was subtracted from the reported informal care time. Results without this weekly time cap are presented as a sensitivity analysis to facilitate comparisons with other studies, as recommended by Oliva-Moreno and colleagues [12].

Out-of-pocket costs were directly measured in monetary terms in the survey. The monthly out-of-pocket cost was assumed to be the midpoint of the reported monthly range, or €300 for those who reported expenses in the €300 or more category. This figure was multiplied by the number of months of care provided to generate annual out-of-pocket costs. Missing values were imputed with the minimum value of less than €50.

#### Indirect (productivity) costs

The study identified three distinct types of care-related absenteeism: Prolonged Time Off (PTO), Temporary Time Off (TTO), and Reduced Hours at Work (RHW). PTO refers to when caregivers discontinue their employment due to the exigencies of providing care. On the other hand, TTO pertains to temporary time off from work for the same reasons. RHW encompasses the reduction in working hours for caregivers who retained their employment. Productivity loss can also come in the form of presenteeism, which refers to diminished on-the-job productivity due to care provision. Indirect (productivity) costs were estimated only for the sub-sample of caregivers aged 16–75 years who reported one or more of these types of productivity loss. The unit cost of productivity loss, i.e., the value of one hour of forgone paid work, was set at the gender-specific hourly labour costs obtained from the Dutch Costing Manual [28, 35].

Two methods were used to estimate indirect (productivity) costs: the human capital approach and the friction cost approach. In the human capital approach, weekly PTO costs were estimated as the number of work hours per week multiplied by the unit cost of productivity loss. The weekly costs were then annualised by multiplying them by the number of work weeks per year in the Netherlands (assumed to be 46 weeks per year). Given that PTO costs are by definition applicable only to non-working caregivers, the number of work hours per week was imputed from the age- and gender-specific national average working hours in 2019, as obtained from Statistics Netherlands [36]. Annual TTO costs were estimated as the product of the reported work hours per week, the duration of time off work (in weeks), and the unit cost of productivity loss. Since the exact duration of time off work was not recorded, it was assumed to correspond with half the number of weeks of care provided in the year preceding the survey. This assumption mirrors the pattern observed in Dutch unemployment data, indicating that the median duration of unemployment spanned half of the working year in 2019 [37]. Weekly RHW costs were estimated as the product of the unit cost of productivity loss and the mean difference in working hours per week between non-caregivers and caregivers who reduced their working hours (see Tables 2 and 3). Weekly RHW costs were annualised in the same fashion as PTO costs, with a minor adjustment for caregivers who had TTO costs. The number of work weeks per year for these caregivers was adjusted by deducting the time off work to avoid double counting.

**Table 2:** Weighted and unweighted descriptive statistics

Characteristic	Unweighted sample (N = 12 348)			Weighted sample (N = 14 222 458)		
	Caregiver, N = 4483	Non-caregiver, N = 7865	p-value^a^	Caregiver, N = 5 074 358	Non-caregiver, N = 9 148 100	p-value^b^
Age in years, Mean (SD)	52.72 (17.06)	50.97 (20.40)	< 0.001	49.65 (17.04)	47.48 (20.11)	< 0.001
Gender, n (%)			< 0.001			< 0.001
Male	1931 (43%)	3974 (51%)		2 261 174 (45%)	4 767 833 (52%)	
Female	2552 (57%)	3891 (49%)		2 813 183 (55%)	4 380 268 (48%)	
Relationship status, n (%)			< 0.001			< 0.001
Married	2775 (62%)	3938 (50%)		2 789 782 (55%)	3 961 995 (43%)	
Divorced	368 (8%)	676 (9%)		499 268 (10%)	824 802 (9%)	
Widow	191 (4%)	590 (8%)		187 921 (4%)	620 002 (7%)	
Never been married	1149 (26%)	2661 (34%)		1 597 386 (31%)	3 741 302 (41%)	
Education, n (%)			< 0.001			< 0.001
Primary	573 (13%)	1356 (17%)		613 827 (12%)	1 470 203 (16%)	
Secondary	2071 (46%)	3310 (42%)		2 387 569 (47%)	3 850 790 (42%)	
Tertiary	1678 (37%)	2713 (34%)		1 907 523 (38%)	3 345 681 (37%)	
None of these	161 (4%)	486 (6%)		165 438 (3%)	481 426 (5%)	
Employment status^c^, n (%)			< 0.001			< 0.001
Not employed	1117 (27%)	1956 (29%)		1 161 678 (24%)	2 016 908 (25%)	
Part-time	1740 (42%)	2487 (36%)		2 028 172 (42%)	3 073 685 (37%)	
Full-time	1281 (31%)	2394 (35%)		1 608 936 (34%)	3 131 751 (38%)	
Not in the labour force^d^	345	1028		275 571	925 757	
Hours of work per week^e^, Mean (SD)	29.61 (12.86)	30.73 (13.78)	< 0.001	29.99 (12.97)	31.08 (13.57)	< 0.001

^a^Wilcoxon rank sum test; Pearson’s chi-squared test

^b^Wilcoxon rank-sum test for complex survey samples; chi-squared test with Rao & Scott’s second-order correction

^c^Employment status refers to the employment status of the respondent during the year prior to the survey. Working full-time is defined as working 36 or more hours per week, and working part-time is defined as working less than 36 h per week

^d^Individuals aged 76 years or older were considered out of the labour force

^e^The number of working hours of employed respondents (n = 3021)

**Table 3:** Summary of cost items

Cost item	N	Unweighted sample (N = 4483)	Weighted sample (N = 5 074 358)
**Direct cost items**			
Hours of care per week (uncapped)	4483		
Mean (SD)		7.98 (17.06)	7.65 (16.42)
Median		3	3
Range		1–168	1–168
Hours of care per week (capped)	4483		
Mean (SD)		7.68 (14.62)	7.37 (14.06)
Median		3	3
Range		1–126	1–126
Hours of care per week (capped), n (%)	4483		
Less than 5 h		2838 (63%)	3 277 688 (65%)
Between 5 and 15 h		1179 (26%)	1 296 502 (26%)
More than 15 h		466 (10%)	500 168 (10%)
Duration of informal care (weeks per year)			
Mean (SD)	4483	41.40 (17.44)	40.93 (17.68)
Median		52	52
Range		1–52	1–52
Monthly out-of-pocket costs, n (%)	3951		
No out-of-pocket costs		2294 (58%)	2 634 969 (59%)
Less than €50		810 (21%)	905 920 (20%)
€50–€100		503 (13%)	559 290 (12%)
€100–€200		190 (5%)	214 554 (5%)
€200–€300		70 (2%)	74 629 (2%)
Greater than €300		84 (2%)	87 987 (2%)
Missing		532	
**Indirect (productivity) cost items**			
Prolonged cessation of work, n (%)	1240^a^		
No		1161 (94%)	1 218 962 (93%)
Yes		79 (6%)	89 962 (7%)
Temporary cessation of work, n (%)	2794^b^		
No		2633 (94%)	3 166 113 (94%)
Yes		161 (6%)	188 402 (6%)
Reduction of working hours, n (%)	2917^c^		
No		2627 (90%)	3 159 877 (90%)
Yes		290 (10%)	341 883 (10%)
Hours of work per week, Mean (SD)	2917^c^		
Caregivers who reduced work hours		28.41 (12.43)	28.93 (12.48)
Caregivers who did not reduce work hours		30.02 (12.73)	30.44 (12.82)
Distracted at work due to caregiving, n (%)	2917^c^		
Rarely or never		2153 (76%)	2 609 977 (77%)
Monthly		250 (9%)	286 275 (8%)
Weekly		296 (10%)	353 595 (10%)
Daily		137 (5%)	157 372 (5%)
Missing		81	
Less concentrated at work due to caregiving, n (%)	2917^c^		
No, not at all		2195 (77%)	2 625 759 (77%)
Yes, a bit		581 (20%)	714 946 (21%)
Yes, very much		60 (2%)	69 662 (2%)
Missing		81	

Note. Percentages may not add up to 100% due to rounding. Indirect (productivity) cost items were assessed only among subjects aged 16 to 75 years who combined work and care in the year preceding the survey

^a^This sample refers to subjects who were not employed at the time of the survey

^b^This sample refers to subjects who were employed at the time of the survey

^c^This sample refers to subjects who had worked for pay during the year preceding the survey

The same calculation procedures were followed for the friction cost approach; however, productivity losses were confined to a friction period of 12 weeks, as per the guidance in the Dutch Costing Manual [28]. While the human capital approach can yield estimates of productivity losses that are many times larger than those from the friction cost approach [38], our study is unlikely to exhibit such marked discrepancies. This prediction stems from the one-year horizon of our research, which limits the human capital approach to assessing potential productivity loss over this one-year timeframe. This constraint, as opposed to the approach’s capability to span a more extended, multi-year caregiving period, is expected to narrow its divergence from the friction cost approach.

To determine the cost of presenteeism, we made certain assumptions to convert the level of reduced concentration resulting from caregiving into a percentage reflecting work impairment. The levels of reduced concentration were converted as follows: “No, not at all” = 0%, “Yes a bit” = 25%, and “Yes, very much” = 50%. Moreover, the frequency of caregiving-related distractions at work was transformed into a percentage of working days affected by such distractions per week. Based on a standard working month of four weeks, each with five working days, the frequencies were converted as follows: “Daily” = 100%, “Weekly” = 20%, “Monthly” = 5%, and “Rarely or never” = 0%. Weekly presenteeism hours were calculated as the product of the percentage of work impairment, the percentage of working days with distraction, and the reported weekly number of hours worked. The resulting figure was then annualised and monetised using an analogous approach as for RHW. Participants with missing values for distraction or reduced concentration at work were assumed to have no presenteeism costs. Given the difficulty in applying the friction cost approach in the context of presenteeism, the associated cost was estimated solely using the human capital approach. As presenteeism was not measured using a standardised instrument, we opted to omit the corresponding costs from the base-case analysis and present them as part of a sensitivity analysis.

#### Total annual societal cost

The total annual societal cost of informal care was estimated in three successive steps, drawing on the guide for estimating informal care costs developed by Landfeldt et al. [39]. The first step involved calculating the annual direct and indirect (productivity) costs for each caregiver in the sample, as previously described. In the second step, indirect (productivity) costs were adjusted to reflect the net societal productivity loss when informal care time was valued using the proxy good method [39].

The proxy good method takes a societal perspective in that it regards informal care as a form of work that, similar to market work, uses time and energy to generate services of economic value [40, 41]. This view implies that shifting time from market activities to informal caregiving does not constitute a loss of productive time but rather a reallocation of labour time from one work activity to another [42]. Nonetheless, such reallocation can still yield net cost if the societal value of the resulting market production losses outweighs that of the production gains from the informal care provided [39]. Therefore, in this analysis, we deducted the cost of informal care time for each caregiver (as measured by the proxy good method) from their indirect (productivity) costs to arrive at their net indirect (productivity) cost.

The literature presents conflicting views on the need for such cost adjustment when employing the opportunity cost method. Some contend that this method inherently adopts the perspective of the informal caregiver, valuing their care time at the forgone value of their best alternative use of that time [43]. Therefore, in cases like our study, where leisure is deemed the best alternative, the assumption of a trade-off exclusively between informal care and leisure activities ensues, obviating the need for adjustment [39, 40]. This is because leisure activities, unlike market work, do not directly contribute to producing goods or services and, therefore, do not need to be factored into estimating indirect (productivity) costs [44]. Other scholars suggest a cautious approach, proposing that informal care time be adjusted by deducting lost market work time. This adjustment is to avoid potential double counting of time that might be reallocated between these two activities [45, 46]. Reflecting these divergent perspectives, we reported the results of the opportunity cost method without adjustment in our base-case analysis and included the adjusted results in a sensitivity analysis.

Finally, we calculated the total cost for each individual in the sample by summing their direct and indirect (productivity) costs. We then aggregated these figures using survey weights to arrive at a nationally representative estimate of the total cost of informal care in the Netherlands.

### Sensitivity analysis

The base-case analysis represents the most conservative estimation of informal care costs. Deterministic one-way sensitivity analyses were performed to gauge the sensitivity of the results to the assumptions applied and the values of key parameters. This included applying alternative unit costs for leisure time. In particular, we applied the willingness-to-pay and willingness-to-accept values for leisure time suggested by Verbooy et al. for the Netherlands [47]. We also assessed the stability of our results with respect to the 126-h weekly cap applied to the time available for work and informal care by recalculating the costs without this cap.

Furthermore, we quantified the potential overestimation in our opportunity cost base-case estimates due to the potential overlap between productivity losses and informal care time. The analysis recalculated the opportunity costs using an adjusted measure of informal care time. This adjustment involved deducting caregivers’ lost paid-work time from their reported informal care time. Finally, we calculated the costs associated with presenteeism using the human capital approach, as described in the Indirect (productivity) costs section.

## Results

### Sociodemographic characteristics and cost variables

Table 2 shows the weighted and unweighted distribution of the main sociodemographic characteristics of survey participants, stratified by caregiver status. The final study population comprised 12 348 individuals, representing a weighted population of 14 222 458 noninstitutionalised adults (≥ 16 years) living in the Netherlands in 2019. Of the total sample, 4483 participants were identified as informal caregivers and included in the subsequent analysis. Our results revealed statistically significant differences between the caregiver and non-caregiver groups in all characteristics examined. Compared to non-caregivers, caregivers tended to be older, female, married, and highly educated. While labour force participation rates were similar between the two groups, working caregivers were more likely to work part-time and fewer hours than their non-caregiver counterparts. Using survey weights, we estimated that there were approximately 5.1 million adult informal caregivers in the Netherlands in 2019, with a prevalence rate of approximately 36% among the adult population. The distribution of the main characteristics of the caregiver sample by gender is presented in Table 7 in Appendix 2.

Table 3 presents a summary of the identified cost items. In terms of the intensity of informal care, the mean weekly hours of care provided was 7.37, and the mean number of weeks of care per year was 40.93. Based on these figures, we estimated that approximately 1.55 billion hours of informal care were provided in the Netherlands in 2019. Moreover, more than 40% of caregivers reported out-of-pocket expenses, with 9% incurring monthly costs greater than €100.

The impact of caregiving on employment was assessed only for the subsample of informal caregivers aged 16 to 75 years who combined work and care in the year preceding the survey. Only 7% of the non-employed respondents in this subsample reported that they had discontinued work due to caregiving duties. Of those employed at the time of the survey, approximately 6% left work temporarily to provide care. Among those who worked for pay in the year before the survey, 10% reported a decrease in their weekly working hours, with an average reduction of approximately two hours compared to non-caregivers. Additionally, nearly one-quarter of those who worked for pay reported experiencing impaired concentration (23%) or distraction at work (23%) due to their caregiving responsibilities. A weighted summary of cost items stratified by gender is presented in Table 8 in Appendix 2.

### Base-case results

#### Opportunity cost method

Table 4 presents the annual costs of informal care in 2019, as calculated using the opportunity cost method. The total cost of informal care time was estimated at €12.41 billion, corresponding to an average annual cost of €2445 per informal caregiver. The out-of-pocket expenses incurred by informal caregivers amounted to €1.57 billion, averaging €309 per caregiver annually.

**Table 4:** Total annual costs of informal care based on the opportunity cost method

Domain	Cost item	Human capital approach				Friction cost approach			
		Costs (€)	Mean (€)	Share of total costs, %	Female-to-male cost ratio	Costs (€)	Mean (€)	Share of total costs, %	Female-to-male cost ratio
A. Direct costs of informal care									
A.1	Informal care time	12 406 021 969	2445	57%	1.36	12 406 021 969	2445	71%	1.36
A.2	Out-of-pocket expenses	1 569 367 944	309	7%	1.10	1 569 367 944	309	9%	1.10
*Sub-total*		13 975 389 913	2754	64%	1.33	13 975 389 913	2754	80%	1.33
B. Indirect (productivity) costs									
B.1	Reduction of work hours	722 361 121	142	3%	1.43	275 211 711	54	2%	1.29
B.2	Temporary cessation of work	3 762 200 914	741	17%	0.83	2 145 917 683	423	12%	0.81
B.3	Prolonged cessation of work	3 416 342 780	673	16%	0.76	1 101 077 929	217	6%	0.76
*Sub-total*		7 900 904 815	1556	36%	0.84	3 522 207 323	694	20%	0.83
**Total societal costs**		21 876 294 728	4310		1.13	17 497 597 236	3448		1.21

Note: Percentages may not add up to 100% due to rounding

The friction cost approach estimated the total annual indirect (productivity) costs to be €3.52 billion, with an average annual cost of €694 per caregiver. Meanwhile, the human capital approach produced a higher estimate of indirect (productivity) costs (by a ratio of 2.24), totalling €7.90 billion, with an average annual cost of €1556 per caregiver. The largest contributor to indirect (productivity) costs was the temporary cessation of work, which accounted for 12%–17% of the total cost.

The aggregate cost of informal care, including both direct and indirect (productivity) costs, was estimated to range from €17.50 billion to €21.88 billion, depending on the method used to estimate indirect (productivity) costs. The cost of informal care time was the most significant cost component, constituting 57%–71% of the total cost, followed by indirect (productivity) costs (20%–36%) and out-of-pocket costs (7%–9%).

The gender-specific analysis highlighted disparities in the economic impact of informal caregiving. The female-to-male cost ratios for informal care time and out-of-pocket expenses were 1.36 and 1.10, respectively, indicating a greater share of costs borne by female caregivers in both categories. Indirect (productivity) costs also carried gender distinctions. The ratio for reduced work hours stood at 1.29–1.43, pointing to higher costs incurred by female caregivers. In contrast, the ratios for temporary and prolonged work cessation were significantly below one, implying higher costs borne by male caregivers. These trends were consistent across both the human capital and friction cost approaches. A breakdown of results by gender is presented in Table 9 in Appendix 2.

#### Proxy good method

Using the proxy good method (see Table 5), we estimated the annual cost of informal care time at €23.42 billion, with an average annual cost of €4615 per informal caregiver. The annual indirect (productivity) costs were estimated to range from €1.70 billion to €5.15 billion using the friction cost and human capital approaches, respectively. As expected, the proxy good method yielded considerably lower estimates for indirect (productivity) costs compared to the opportunity cost method. This outcome was due to the production gains from reallocating time from paid labour to informal care, which partially offset the indirect (productivity) costs.

**Table 5:** Total annual costs of informal care based on the proxy good method

Domain	Cost item	Human capital approach				Friction cost approach			
		Costs (€)	Mean (€)	Share of total costs, %	Female-to-male cost ratio	Costs (€)	Mean (€)	Share of total costs, %	Female-to-male cost ratio
A. Direct costs of informal care									
A.1	Informal care time	23 420 148 790	4615	78%	1.36	23 420 148 790	4615	88%	1.36
A.2	Out-of-pocket expenses	1 569 367 944	309	5%	1.10	1 569 367 944	309	6%	1.10
*Sub-total*		24 989 516 734	4924	83%	1.34	24 989 516 734	4924	94%	1.34
B. Indirect (productivity) costs									
*Sub-total*		5 145 262 608	1014	17%	0.79	1 700 520 930	335	6%	0.79
**Total societal costs**		30 134 779 342	5938		1.23	26 690 037 664	5259		1.30

Note: Percentages may not add up to 100% due to rounding

The total annual costs of informal care ranged between €26.69 billion and €30.13 billion (equivalent to €5259–€5938 per caregiver), depending on whether the friction cost or human capital approach was used to estimate the indirect (productivity) costs. Informal care time costs constituted 78%–88% of the total costs, while indirect (productivity) and out-of-pocket costs accounted for 6%–17% and 5%–6%, respectively.

The gender-specific analysis based on the proxy good method yielded results that echoed those of the opportunity cost method, with similar gender disparities observed across all cost categories. Detailed results by gender are shown in Table 10 in Appendix 2.

#### Sensitivity analysis results

The sensitivity analysis demonstrated that the unit cost of leisure time greatly impacted the cost estimates. Using the willingness-to-pay value for leisure time proposed by Verbooy et al. resulted in annual costs ranging from €21.08 billion to €25.46 billion [47]. These estimates represented increases of 16.39% and 20.50% compared with the base-case estimates obtained with the opportunity cost method. Employing the willingness-to-accept value yielded even higher cost estimates (€31.62 billion–€36.00 billion), indicating increases of 64.57% and 80.73% above the base-case estimates.

Applying the 126-h weekly cap minimally affected the cost estimates. Omitting this cap resulted in increases of 2.01%–2.51% from the opportunity cost base-case estimates and 2.75%–3.11% from the proxy good base-case estimates. This meagre effect was expected, considering the limited number of cases that required the cap.

The adjustment of informal care time, which involved deducting lost paid-work time, led to a modest reduction in the base-case estimates of total opportunity cost. The analysis placed the potential overestimation at 3.20% or 4.55%, depending on whether the friction cost or human capital approach was used to calculate indirect (productivity) costs (see Table 11 in Appendix 3).

Finally, the estimated cost of presenteeism among caregivers in 2019 was €1.79 billion (M = €352). When included in the proxy good estimate, the total cost increased by 5.93%. The same figure rose to 8.17% when presenteeism was factored into the opportunity cost estimate, contributing to 7.55%–18.45% of the total and indirect (productivity) costs, respectively. None of the sensitivity analyses significantly distorted the distribution of cost components in either the opportunity cost method or the proxy good method.

## Discussion

### Main findings

Our study estimated the number of adult informal caregivers in the Netherlands at 5.1 million in 2019, indicating a prevalence rate of approximately 36% among the country’s adult population. These estimates align with previous research and suggest a 16% increase in the number of caregivers and a 4-percentage point increase in prevalence since 2016 [48]. A significant factor contributing to this trend is the country’s rapidly expanding population of older adults (≥ 65 years), which grew by 7.4% between the two years [49].

Furthermore, this study estimated the total costs of informal care in the Netherlands in 2019 to range between €17.50 billion and €30.13 billion, depending on the methods used to estimate informal care time and indirect (productivity) costs. These figures highlight the substantial economic burden of informal care on Dutch society. To contextualise these figures, the estimated annual cost of informal care in 2019 accounts for 2.15%–3.71% of Dutch GDP in the same year (€813 billion). Interestingly, these costs are of a similar magnitude to those of public spending on LTC (2.67% of GDP) and nearly half of that on education (5.20% of GDP) [50, 51]. To put these economic figures into individual terms, if the Dutch adult population were to shoulder the burden of informal care costs equally, an annual contribution of €1230 to €2119 per person would be required. Should these costs be apportioned through a uniform increase in income tax for all working adults in the Netherlands, their annual tax payments could rise by approximately €1778 to €3062. This increment would raise the tax burden by a margin equivalent to 5%–8% of the national average annual gross income [52].

The study also estimated that Dutch informal caregivers provided approximately 1.55 billion hours of care in 2019, equivalent to 842 416 full-time working years (assuming 1836 working hours per FTE). The economic value of this care was substantial, ranging from €12.4 billion to €23.4 billion, making it the primary contributor to informal care costs. Female caregivers accounted for approximately 58% of these costs, reflecting their provision of more caregiving hours than their male counterparts—a consistent finding in informal care research [8, 22]. This disparity likely stems from longstanding societal and cultural norms that traditionally assign caregiving roles primarily to women [53]. In the Dutch context, the labour market dynamics likely contribute further to this trend. More than 70% of employed Dutch women work part-time [54], potentially affording them greater flexibility and more time for caregiving [55, 56].

Care-related out-of-pocket expenses in the Netherlands are notably modest. Dutch caregivers spent an average of €309 annually, significantly less than the €5363 (PPP- and inflation-adjusted to 2019 Euro) reported for their American counterparts [57]. This low out-of-pocket expenditure in the Netherlands may be attributed to available supportive measures, including comprehensive health insurance and pension plans, financial support (e.g., personal budget), and home care services [58, 59]. Such measures can alleviate financial burdens on care recipients and their caregivers, particularly in areas of high expenditure, such as housing adaptations, healthcare services, specialised aids, and transportation [60]. Our analysis also reveals slight gender-based disparities in out-of-pocket expenses. While female caregivers collectively incur higher expenses, the mean individual expenditure for their male counterparts is about 13% higher (see Table 9 in Appendix 2). This difference may reflect societal norms that tend to cast men as primary financial providers, potentially tilting their contributions towards financial support [61]. Furthermore, the existing wealth and income gap, favouring men [62], seemingly facilitates their ability to shoulder these expenses. Considering the ongoing trend towards ageing in place and the anticipated rise in the number of individuals needing care, it becomes essential for future studies to continue examining these out-of-pocket expenditures.

Moreover, the study estimated the market productivity loss attributed to informal caregiving to range from $1.7 billion to $7.9 billion, depending on the valuation method. This loss corresponds to an estimated 45.1 million to 210.3 million hours of paid labour forgone, tantamount to the productivity loss of 24 568 to 114 537 full-time jobs in 2019 (assuming 1836 working hours per FTE). In examining the different forms of this productivity loss, we found that only 7% of non-working caregivers quit their previous jobs due to caregiving responsibilities. This finding is consistent with previous research demonstrating a modest negative correlation between labour force participation and informal care provision [63, 64]. Despite the relative infrequency of workforce exit among caregivers, it still carries a considerable economic burden. We estimated this burden to be between €1.10 billion and €3.42 billion, rendering it the second largest driver of indirect (productivity) costs (31%–43% of total indirect costs). The short time horizon of our study precluded accounting for the potential long-term societal costs of workforce exit. However, it is imperative to emphasise that withdrawal from the workforce, even temporarily, can have significant long-term consequences [65]. For example, caregivers who discontinue paid employment may face challenges when reintegrating into the labour market, such as overcoming gaps in employment history and addressing skill depreciation. These challenges can ultimately lead to reduced future employability and earning potential [66]. Consequently, further research is warranted to understand the long-term implications of workforce exit among caregivers and identify policy measures that can assuage these costs.

Despite being the most prevalent form of productivity loss, reducing work hours accounted for only a minor portion (8%–9%) of the total indirect (productivity) costs. Notably, the costs associated with this arrangement were 29%–43% higher for female caregivers, likely reflecting their greater involvement in part-time employment. This mode of employment offers the flexibility to adjust work hours in response to escalating caregiving demands rather than leaving the workforce [67, 68].

By contrast, temporary leave emerged as the primary driver of indirect (productivity) costs, comprising 48%–61% of these costs. When combined with leaving the workforce entirely, the total contribution to indirect (productivity) costs exceeded 90%. Remarkably, these costs were disproportionately higher for male caregivers, who faced 20%–31% higher costs than their female counterparts. This difference is partly attributable to the application of national gender-specific hourly labour costs, which are higher for men, in correspondence with the gender wage gap in the Dutch labour market [62]. Additionally, the average working hours for men are substantially higher, mainly owing to the higher incidence of full-time employment [36, 54]. Furthermore, the rigidity of traditional full-time employment arrangements limits men’s capacity to adjust their working hours to meet caregiving needs [69]. Consequently, male caregivers with increasing caregiving responsibilities are more likely to be forced out of employment than their female counterparts [67]. This situation contrasts with women’s experiences, who more often engage in part-time work and thus have the option of a more gradual reduction in work hours when balancing employment and caregiving duties.

This breakdown of indirect (productivity) costs suggests that reducing work schedules, instead of withdrawing from the workforce, can offer a mutually beneficial solution for caregivers and their employers. In addition to curtailing productivity loss, continuing to work, even with reduced hours, can bolster caregivers’ emotional and social well-being [70]. Employers can also capitalise on this arrangement by retaining skilled employees, preserving institutional knowledge, maintaining workflow, and reducing recruitment and training expenses [71, 72].

In the Netherlands, national policies such as the Work and Care Act and the Flexible Work Act facilitate the balance between work and caregiving responsibilities [73]. The latter enables employees to request flexible working conditions, including changes in work hours or locations [74]. The Work and Care Act provides informal caregivers with short-term and long-term care leaves to assist a sick person. Short-term care leave extends to twice the caregiver’s weekly working hours, remunerated at 70% of their salary with a minimum wage guarantee. Long-term care leave, typically unpaid, can extend up to six times the caregiver’s weekly hours [75]. However, the utilisation of these leaves, particularly the long-term care leave, remains significantly limited [76].

To further support informal caregivers in maintaining their employment, we propose expanding care leave in terms of duration, remuneration, and coverage. For example, in Belgium, unlike in the Netherlands, self-employed caregivers can receive care benefits for up to 12 months [77]. Italy offers caregivers generous short-term (3 days per month) and long-term (up to 2 years) paid care leaves, with full salary and pension contributions [78]. Additionally, new forms of flexible work arrangements, such as flexible weekly scheduling, team scheduling, and shift swapping, can accommodate the unpredictable and episodic nature of caregiving [79]. These arrangements are particularly beneficial for caregivers with lower wages, whose roles are often more rigidly tied to time and location [80]. Lastly, it is crucial to raise awareness about existing work-care policies and flexible work arrangements, emphasising the value they bring to both caregivers and organisations.

### Comparisons with other studies

Our research findings concur with those of analogous studies in different countries. However, caution is warranted when drawing such comparisons due to disparities in definitions of informal care, target demographics, methodologies, and cost components evaluated.

Internationally, our findings align with studies conducted in the United States and Australia. These studies deduce that informal care costs constitute up to 4% of GDP in both countries [16, 18]. This finding is consistent with our results for the Netherlands, underscoring the significant economic impact of informal care in various high-income countries despite differences in LTC systems.

A comparison with other European studies reveals both divergences and parallels. For example, the study by Ekman and colleagues estimates the opportunity cost of informal care in Sweden at 3.15% of GDP, which is 17% higher than our analogous figure for the Netherlands (derived using the human capital approach) [20]. This discrepancy is primarily due to their inclusion of factors such as presenteeism and disturbed sleep, which are not considered in our base-case analysis. Excluding these factors, Sweden’s estimate decreases to 2.63% of GDP, nearly matching our estimate of 2.69%. Conversely, the Spanish study by Oliva-Moreno et al. reports costs per caregiver 10 to 17 times higher than in the Netherlands, signifying pronounced variations in care intensity consistent with the North–South informal care usage gradient in Europe [19, 81]. The Irish study by Hanly and Sheerin offers a gender-specific analysis, showing that women account for two-thirds of informal care costs [22]. This proportion is somewhat higher than in our study, where women account for about 58% of caregiving time costs. This gender disparity in our study diminishes when indirect and out-of-pocket expenses are considered, suggesting a relatively more equitable gender distribution of informal care costs in the Netherlands. The Dutch context, characterised by more developed formal care and higher part-time employment rates among women, seems to somewhat alleviate the informal care load and lessen the employment-care trade-offs for women.

Finally, the Ecorys report offers a direct comparison within the Netherlands [21]. Their €22 billion estimate, based on the opportunity cost method, initially appears congruent with our upper-bound estimate. However, a detailed analysis uncovers notable differences. The Ecorys estimate accounts for a subset of indirect (productivity) costs, specifically those related to reduced work hours, while our estimate encompasses a more comprehensive range of indirect costs. Aligning our estimate with the Ecorys methodology results in a €14.7 billion figure, approximately 33% lower than the Ecorys estimate. A key strength of our study lies in using individual-level data, allowing for a precise estimation of the full spectrum of informal care costs. On the contrary, the Ecorys report relied exclusively on data from published reports and articles, which did not offer sufficient detail to capture the full scope of informal care costs. Furthermore, our study adheres to the Dutch guidelines for economic evaluations in healthcare, ensuring the comparability and interpretability of our findings [82, 83]. Specifically, we applied the friction cost approach to estimate indirect (productivity) costs and sourced relevant unit costs from the Dutch costing manual [28].

### Limitations

This study has certain limitations that should be acknowledged. First, the study did not account for the non-economic costs and benefits that may result from changes in caregivers’ physical, social, and emotional well-being [84]. Although recent literature increasingly focuses on these costs and benefits [13, 85], they are still often omitted from cost analyses due to challenges in measurement and valuation [86, 87]. While the importance of these costs and benefits cannot be overstated, we could not incorporate them due to a paucity of pertinent data.

The study also excluded transaction costs associated with informal care transfers (e.g., personal budget). Although these transfers do not represent real costs to society, they generate transaction costs that warrant consideration [88, 89]. However, estimating these costs is a formidable challenge due to the complex network of stakeholders involved, including municipalities, health insurers, and intermediaries responsible for implementing payments [90]. These transaction costs are also unlikely to constitute a significant portion of overall informal care costs. Therefore, we chose to omit them from our analysis.

Another aspect not covered in our study is the health care costs associated with informal caregiving. While informal caregiving can negatively affect caregivers’ physical and mental health, evidence of its impact on health care utilisation is limited and inconsistent [91, 92]. For instance, a study by Shaffer and Nightingale using nationally representative data from the United States found no significant differences in the frequency or number of health care appointments between informal caregivers and non-caregivers [93]. Another study by Van Houtven et al. showed higher drug utilisation rates among caregivers who provided intensive care to older people with dementia [94]. However, the study concluded that the impact on informal care costs was minor and unlikely to have significant implications.

Another limitation relates to the measurement of informal care time. Research has indicated that informal caregivers may overestimate their care time [39]. This measurement bias can arise from the joint production of informal care and normal housework or the difficulty in distinguishing between the two [15]. Despite the survey’s instruction to exclude time spent on normal housework and usual care for housemates when reporting informal care time, some caregivers may have struggled to distinguish between these activities. This issue may have been particularly pronounced for caregivers living with the care recipient, providing high-intensity care, or caring for an extended period.

A further limitation lies in our approach to estimating out-of-pocket costs. Specifically, we capped these costs at €300 per month for the highest expense bracket due to the categorical nature of our data. This approach may have led to underestimating out-of-pocket costs for informal caregivers whose expenditures significantly exceeded this threshold. However, only 2% of caregivers reported expenses above the €300 cap. Therefore, while the potential for underestimation exists, its impact on our overall findings is likely minimal.

The final limitation pertains to the estimation of presenteeism costs. This estimation was based on responses to two survey questions about the frequency of distraction and the level of impaired concentration experienced by informal caregivers at work. We did not use standardised instruments such as the iMTA Productivity Cost Questionnaire [95] or the Caregiver Indirect and Informal Care Cost Assessment Questionnaire [39]. Consequently, our estimates for presenteeism costs should be interpreted cautiously, and they are presented separately from other costs in recognition of this limitation.

## Conclusion

The shift towards LTC models in which informal caregivers play an increasingly larger role represents a significant change that demands careful consideration of its implications. While this change may appear fiscally responsible, our analysis indicates that it could carry substantial external costs borne by stakeholders and sectors beyond the intended targets of the policy. To our knowledge, this study is the first to provide nationally representative estimates of these external costs associated with informal care in the Netherlands.

We present compelling evidence for the immense value that informal care brings to society. Millions of caregivers make significant personal sacrifices, dedicating billions of euros’ worth of time and resources to providing care. This infusion of resources into the Dutch LTC system effectively bridges existing care gaps by subsidising public-sector care provision. Absent these resources, the Dutch government would face a tremendous challenge in providing care for all those in need.

Despite its significant contributions, informal care remains largely excluded from national income accounts. This exclusion effectively renders these contributions invisible in standard macroeconomic frameworks, relegating informal care to the margins of economic policy discourse. This marginalisation jeopardises the sustainability and quality of informal care and, inadvertently, undermines support for caregivers. Moreover, our findings demonstrate that informal care can adversely affect labour market productivity and supply. This impact reinforces the argument for integrating informal care into the analysis of labour market policy. Such integration could contribute to harnessing caregivers’ potential in the labour market while preserving their invaluable caregiving roles.

The cost estimates we provide here can aid policymakers in adopting a comprehensive approach to the LTC system, which recognises the scarcity and value of all available resources—whether public, private, or informal. Such an inclusive outlook is crucial to inhibiting unproductive cost-shifting and determining the appropriate mix of LTC services that balances financial constraints with social welfare objectives.

## Appendix 1: Unit costs

See Table 6.

**Table 6:** Unit costs of resource use

	Unit of measurement	Unit cost (€)	Source
The opportunity cost of leisure time	Hour	8.02	The average value of one hour of non-business travel time savings for all surface modes [31]
WTP for leisure time	Hour	10.34	[47]
WTA for leisure time	Hour	17.15	[47]
Work productivity loss^a^	Hour	34.18 (Women) 40.99 (Men)	[28]
Household/domestic carer	Hour	15.14	[28]

*Note.* Unit costs are presented in 2019 price levels. *WTP* willingness-to-pay, *WTA* willingness-to-accept

^a^Lost paid-work time

## Appendix 2: Gender-specific analysis

See Tables 7, 8, 9 and 10.

**Table 7:** Weighted and unweighted descriptive statistics by gender for the caregiver sample

Characteristic	Unweighted caregiver sample (N = 4483)			Weighted caregiver sample (N = 14 222 458)		
	Male, N = 1931	Female, N = 2552	p-value^a^	Male, N = 7 029 007	Female, N = 7 193 451	p-value^b^
Age in years, Mean (SD)	53.78 (17.23)	51.91 (16.90)	< 0.001	47.70 (18.81)	48.79 (19.36)	0.008
Relationship status, n (%)			< 0.001			< 0.001
Married	1232 (64%)	1543 (60%)		3 381 312 (48%)	3 370 465 (47%)	
Divorced	144 (7%)	224 (9%)		584 658 (8%)	739 413 (10%)	
Widow	49 (3%)	142 (6%)		199 105 (3%)	608 818 (8%)	
Never been married	506 (26%)	643 (25%)		2 863 932 (41%)	2 474 755 (34%)	
Highest level of education, n (%)			< 0.001			0.001
Primary education	265 (14%)	308 (12%)		1 017 363 (14%)	1 066 667 (15%)	
Secondary education	793 (41%)	1278 (50%)		2 973 607 (42%)	3 264 752 (45%)	
Tertiary education	797 (41%)	881 (35%)		2 724 218 (39%)	2 528 986 (35%)	
None of these	76 (4%)	85 (3%)		313 819 (4%)	333 046 (5%)	
Employment status^c^, n (%)			< 0.001			< 0.001
Not employed or retired	412 (24%)	705 (30%)		1 313 109 (20%)	1 865 477 (29%)	
Part-time	427 (24%)	1313 (55%)		1 630 422 (25%)	3 471 435 (53%)	
Full-time	912 (52%)	369 (15%)		3 564 875 (55%)	1 175 812 (18%)	
Not in the labour force^d^	180	165		520 601	680 727	
Hours of work per week^e^, Mean (SD)	34.97 (13.01)	25.35 (11.01)	< 0.001	34.95 (13.34)	25.90 (11.67)	< 0.001

^a^Wilcoxon rank sum test; Pearson’s chi-squared test

^b^Wilcoxon rank-sum test for complex survey samples; chi-squared test with Rao & Scott’s second-order correction

^c^Employment status refers to the employment status of the respondent during the year prior to the survey. Working full time is defined as working 36 or more hours per week, and working part time is defined as working less than 36 h per week

^d^Individuals aged 76 years or older were considered out of the labour force

^e^The number of working hours of employed respondents (n = 3021)

**Table 8:** Weighted summary of cost items by gender

Cost item	Male, N = 2 261 174	Female, N = 2 813 183
**Direct cost items**		
Hours of care per week (uncapped), Mean (SD)	7.11 (14.50)	8.08 (17.81)
Hours of care per week (capped), Mean (SD)	6.94 (12.86)	7.71 (14.94)
Hours of care per week (capped), n (%)		
Less than 5 h	1 511 820 (67%)	1 765 868 (63%)
Between 5 and 15	526 875 (23%)	769 627 (27%)
Greater than 15	222 479 (10%)	277 689 (10%)
Duration of informal care (weeks per year), Mean (SD)	41.12 (17.56)	40.77 (17.78)
Monthly out-of-pocket costs, n (%)		
No out-of-pocket costs	1 146 038 (57%)	1 488 931 (60%)
Less than €50	399 101 (20%)	506 819 (20%)
Between €50 and €100	259 695 (13%)	299 595 (12%)
Between €100 and €200	100 522 (5%)	114 033 (5%)
Between €200 and €300	36 077 (2%)	38 552 (2%)
Greater than €300	54 922 (3%)	33 064 (1%)
Missing (unweighted)	229	303
**Indirect (productivity) cost items**		
Prolonged cessation of work, n (%)		
No	458 713 (92%)	760 249 (94%)
Yes	39 791 (8%)	50 171 (6%)
Temporary cessation of work, n (%)		
No	1 482 876 (95%)	1 683 237 (94%)
Yes	81 259 (5%)	107 143 (6%)
Reduction of working hours, n (%)		
No	1 487 176 (91%)	1 672 701 (89%)
Yes	139 413 (9%)	202 470 (11%)
Distraction at work, n (%)		
Rarely or never	1 202 902 (74%)	1 407 076 (75%)
Monthly	138 573 (9%)	147 702 (8%)
Weekly	158 296 (10%)	195 298 (10%)
Daily	78 789 (5%)	78 583 (4%)
Refusal	48 029 (3%)	46 512 (2%)
Missing (unweighted)	638	928
Less concentration at work, n (%)		
No, not at all	1 233 372 (76%)	1 392 387 (74%)
Yes, a bit	322 162 (20%)	392 784 (21%)
Yes, very much	31 285 (2%)	38 378 (2%)
Refusal	39 771 (2%)	51 622 (3%)
Missing (unweighted)	638	928

Note. Percentages may not add up to 100% due to rounding. Indirect (productivity) cost items were assessed only among subjects aged 16 to 75 years who combined work and care in the year preceding the survey

**Table 9:** Total annual costs of informal care based on the opportunity cost method by gender

Domain	Cost item	Human capital approach				Friction cost approach			
		Male		Female		Male		Female	
		Costs (€)	Mean (€)	Costs (€)	Mean (€)	Costs (€)	Mean (€)	Costs (€)	Mean (€)
A. Direct costs of informal care									
A.1	Informal care time	5 249 018 308	2321	7 157 003 660	2544	5 249 018 308	2321	7 157 003 660	2544
A.2	Out-of-pocket expenses	748 464 127	331	820 903 817	292	748 464 127	331	820 903 817	292
* Sub-total*		5 997 482 435	2652	7 977 907 477	2836	5 997 482 435	2652	7 977 907 477	2836
B. Indirect (productivity) costs									
B.1	Reduction of work hours	297 817 653	132	424 543 468	151	120 410 803	53	154 800 908	55
B.2	Temporary cessation of work	2 054 409 388	909	1 707 791 525	607	1 183 023 580	523	962 894 103	342
B.3	Prolonged cessation of work	1 940 463 162	858	1 475 879 618	525	625 385 210	277	475 692 719	169
* Sub-total*		4 292 690 203	1898	3 608 214 611	1283	1 928 819 594	853	1 593 387 729	566
Total societal costs		10 290 172 638	4551	11 586 122 088	4119	7 926 302 029	3505	9 571 295 206	3402

Note: Percentages may not add up to 100% due to rounding

**Table 10:** Total annual costs of informal care based on the proxy good method by gender

Domain	Cost item	Human capital approach				Friction cost approach			
		Male		Female		Male		Female	
		Costs (€)	Mean (€)	Costs (€)	Mean (€)	Costs (€)	Mean (€)	Costs (€)	Mean (€)
A. Direct costs of informal care									
A.1	Informal care time	9 909 122 368	4382	13 511 026 422	4803	9 909 122 368	4382	13 511 026 422	4803
A.2	Out-of-pocket expenses	748 464 127	331	820 903 817	292	748 464 127	331	820 903 817	292
* Sub-total*		10 657 586 495	4713	14 331 930 239	5095	10 657 586 495	4713	14 331 930 239	5095
B. Indirect (productivity) costs									
* Sub-total*		2 875 508 307	1272	2 269 754 301	807	949 209 637	420	751 311 293	267
**Total societal costs**		13 533 094 802	5985	16 601 684 540	5902	11 606 796 132	5133	15 083 241 532	5362

Note: Percentages may not add up to 100% due to rounding

## Appendix 3: Sensitivity analysis

See Table 11.

**Table 11:** Total annual costs of informal care based on the opportunity cost method (adjusted informal care time)

Domain	Cost item	Human capital approach			Friction cost approach		
		Costs (€)	Mean (€)	Share of total costs, %	Costs (€)	Mean (€)	Share of total costs, %
A. Direct costs of informal care							
A.1	Informal care time	11 409 925 050	2249	55%	11 845 675 266	2334	70%
A.2	Out-of-pocket expenses	1 569 367 944	309	8%	1 569 367 944	309	9%
* Sub-total*		12 979 292 994	2558	62%	13 415 043 210	2643	79%
B. Indirect (productivity) costs							
B.1	Reduction of work hours	722 361 121	142	3%	275 211 711	54	2%
B.2	Temporary cessation of work	3 762 200 914	741	18%	2 145 917 683	423	13%
B.3	Prolonged cessation of work	3 416 342 780	673	16%	1 101 077 929	217	7%
* Sub-total*		7 900 904 815	1556	38%	3 522 207 323	694	21%
**Total societal costs**		20 880 197 808	4115		16 937 250 533	3337	

Note: Percentages may not add up to 100% due to rounding
